# Recurrent Acquired Angioedema as a Heralding Sign of Splenic Marginal Zone Lymphoma Recurrence

**DOI:** 10.7759/cureus.81047

**Published:** 2025-03-23

**Authors:** Taxiarhia J Arabatzis, Chandrakala J Dadeboyina, Haleigh J Minor, Xiaohong Mary Zhang, Prianka Ray

**Affiliations:** 1 Internal Medicine/Pediatrics, Geisinger Medical Center, Danville, USA; 2 Hematology and Oncology, Geisinger Medical Center, Danville, USA; 3 Anatomic and Clinical Pathology, Geisinger Medical Center, Wilkes-Barre, USA; 4 Hematology and Medical Oncology, Geisinger Medical Center, Danville, USA

**Keywords:** acquired angioedema, hereditary angioedema, lymphoprolierative disorders, splenic marginal zone lymphoma, warm autoimmune hemolytic anemia

## Abstract

Acquired angioedema (AAE) is a rare disorder caused by an acquired deficiency in C1 esterase inhibitor leading to recurrent episodes of potentially life-threatening angioedema and is often associated with underlying lymphoproliferative disorders. Many cases of acquired angioedema are associated with lymphoproliferative disorders. Here, we present a case of a middle-aged woman who presented with multiple episodes of angioedema. She developed hemolytic anemia associated with angioedema, which prompted the diagnosis of an underlying splenic marginal zone lymphoma. Recurrent angioedema, particularly in middle-aged or older patients, should prompt a thorough workup for lymphoproliferative disorders, as timely treatment of the underlying condition can resolve angioedema and prevent life-threatening complications.

## Introduction

Angioedema, or swelling of mucosal, submucosal, or subcutaneous tissue, can be classified as hereditary or acquired [1]. Hereditary angioedema typically presents in the first two decades of life, often with a strong family history. In contrast, acquired angioedema (AAE) usually presents in the fourth decade of life and is caused by overactivation of the classical complement pathway, leading to consumption of the C1 esterase inhibitor (C1-INH), which is a protein that mediates inflammation and vasodilation leading to angioedema [1-4]. In both cases, angioedema is mediated by bradykinin or mast cell mediator release causing vascular permeability of small blood vessels resulting in the clinical manifestation of edema [3]. Angioedema itself is a life-threatening disorder that typically affects three regions: the skin, the mucosal tissues of the upper respiratory and gastrointestinal tracts, and the larynx [5,6].

The prevalence of AAE is difficult to estimate due to its rarity, but it is thought to occur in 1 in 100,000 to 1 in 500,000 patients [4]. Effective treatment of the underlying disease in AAE can resolve angioedema, and if no underlying condition is identified initially, regular screening for associated disease, particularly lymphoproliferative disorders, is essential [1,7]. Missing a diagnosis of angioedema can lead to delays in the recognition and treatment of underlying lymphoproliferative disorders, such as splenic marginal zone lymphoma (SMZL). The exact biological link between acquired angioedema and lymphoproliferative disorders remains unknown and is an active area of research, but the association between them and resolution with angioedema with the treatment of underlying lymphoproliferative disease has been shown [8].

Here we describe a case of a woman who presented in her 50s with multiple episodes of angioedema before being diagnosed with SMZL. She initially responded to treatment with steroids and rituximab. She subsequently developed recurrent angioedema, which heralded a recurrence of her SMZL, for which she was treated with combined rituximab and bendamustine. 

## Case presentation

An adult female in her 50s presented to the emergency department (ED) with swelling of the tongue and face requiring emergent intubation and ICU admission. The following day, she was able to be extubated. Laboratory studies at the time revealed negative serum paraprotein studies, low complement C4, and low C1 inhibitor levels (Table 1). She was diagnosed with angioedema of unknown etiology, but no further work-up was conducted at that time, delaying the identification of an underlying cause. In outpatient, she followed up with an allergist, and lab work at that time showed low levels of C4, C1 esterase inhibitor activity and protein, as well as low CH50 (Table 1). Low levels of C4, C1 esterase inhibitor activity and protein lead to increased release of bradykinin and other vasoactive peptides, which results in symptoms of angioedema. Based on the noted labs she was given a presumed diagnosis of hereditary angioedema. In the next 4 months, she had two additional episodes of angioedema requiring medical attention. Her symptoms were treated, but no additional work-up was done. 

**Table 1:** Immunology Labs

Lab	Reference value	Patient level at first admission	Patient levels as an outpatient
Completed C4	10-40 mg/dl	<2 mg/dl	<2 mg/dl
C1 esterase inhibitor level activity	>= 68%	12%	34%
C1 esterase inhibitor protein	21-39 mg/dl	Not collected	11 mg/dl
CH50	31-60 U/ml	Not collected	< 10 U/ml

**Figure 1:**
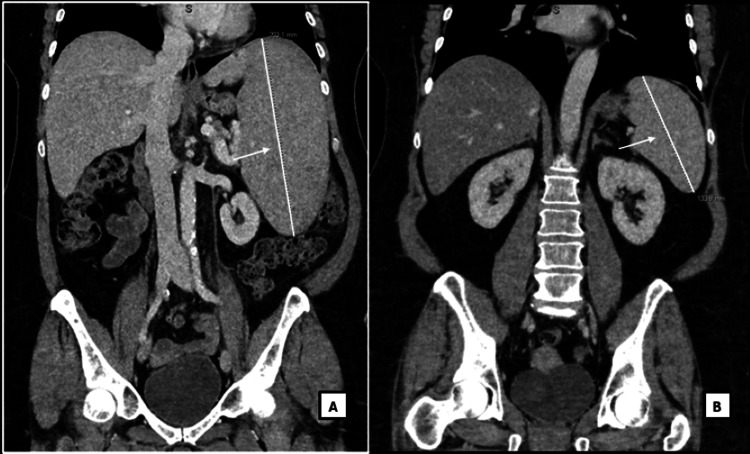
Splenomegaly A. Splenomegaly measuring 20.3 cm in length as shown with white line and white arrow. B. Resolved splenomegaly, spleen measuring 13.4 cm, as shown with white line and white arrow, after rituximab treatment. Improved spleen size is a marker of appropriate response to rituximab.

Almost two years later, she presented with severe symptomatic anemia. She was admitted and a work-up revealed a diagnosis of Coombs-positive warm autoimmune hemolytic anemia with low hemoglobin, high lactate dehydrogenase (LD), low haptoglobin, normal total bilirubin, and a positive direct Coombs IgG protein (Table 2). Full body CT showed massive splenomegaly (20.3 cm in anteroposterior (AP) diameter) and a new splenic infarct (Figure 1A). Serum urine paraprotein studies were negative. She underwent bone marrow biopsy and flow cytometry of the bone marrow and serum. 

**Table 2:** Hemolytic Anemia Labs

Lab	Reference Value	Patient Values
Hemoglobin	12.0-15.3 g/dl	7.1 g/dl
Lactate dehydrogenase (LD)	< = 250 U/l	295 U/l
Haptoglobin	30-200 mg/dl	23 mg/dl
Total Bilirubin	<= 1.2 mg/dl	0.7 mg/dl

**Figure 2:**
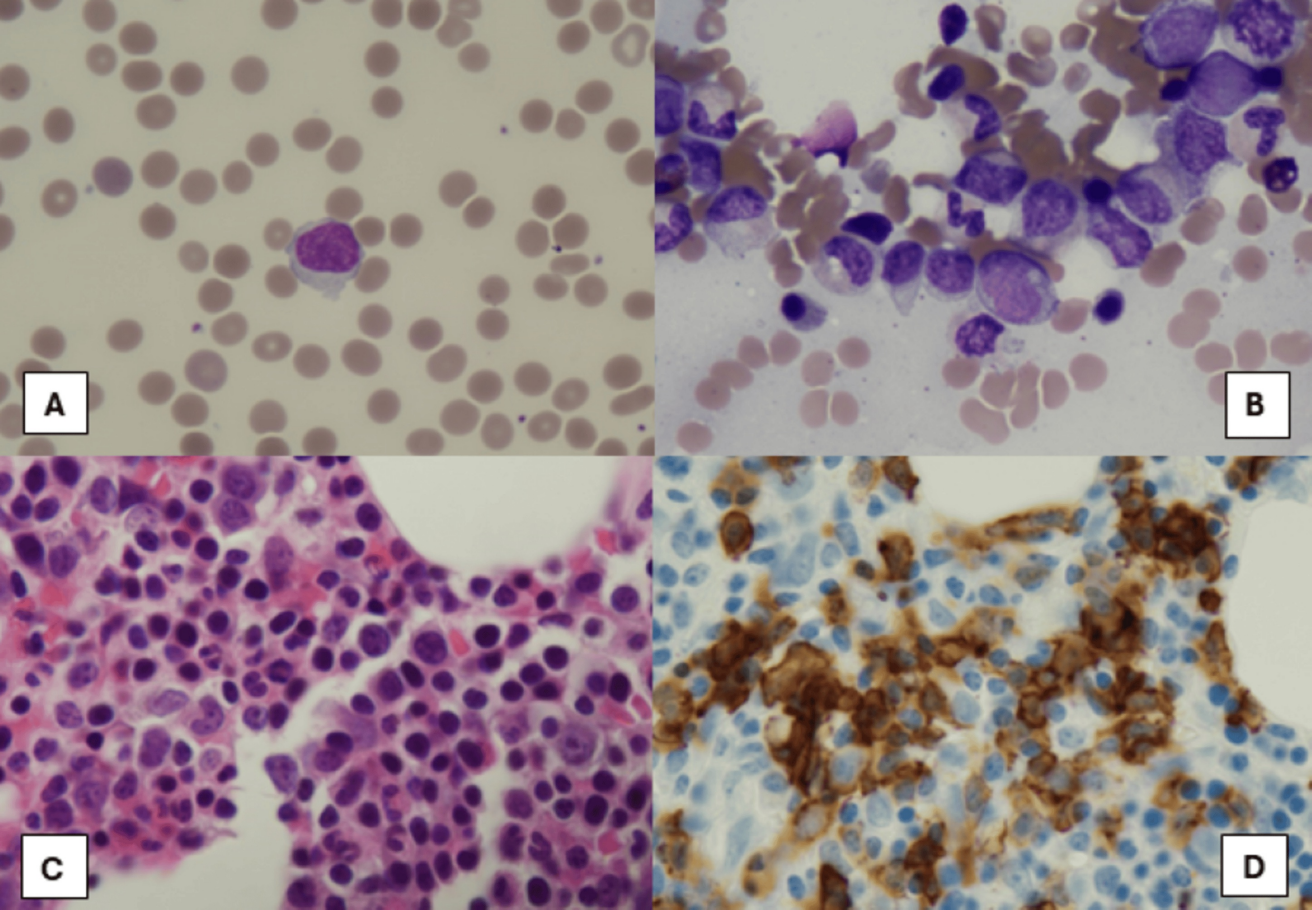
Bone Marrow Pathology Slides A: Peripheral blood smear contains atypical lymphocytes. B: Bone marrow aspirate smears show occasional atypical lymphocytes. C: Bone marrow biopsy with focal atypical lymphocytes. D: Immunohistochemical stain of CD20 highlights atypical/neoplastic lymphocytes. Together, these pathology slides helped lead to a diagnosis of splenic marginal zone lymphoma.

**Figure 3:**
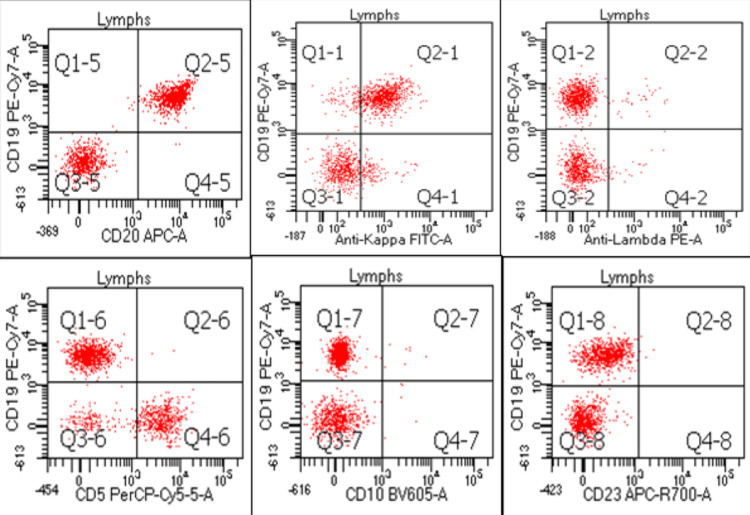
Flow Cytometry Results of Bone Marrow Biopsy A population of monotypic B-cells is identified from bone marrow flow cytometry. The monotypic B cells are positive for CD19 and CD20 with surface immunoglobulin kappa light chain restriction; these B-cells are negative for CD5, CD10, and CD23. Flow cytometry results helped determine the diagnosis of splenic marginal zone lymphoma. This figure was created by the authors using Microsoft Powerpoint (Microsoft Corporation, Redmond, USA).

Bone marrow biopsy showed hypercellular marrow with multilineage maturation (Figure 2A-2D). Mature B cell lymphoma cells were positive for CD19, CD20, and FMC-7 with surface immunoglobin kappa light chain restriction, and negative for CD5, CD10, CD23, and CD43 representing approximately 10-15% of marrow cellularity. Fluorescence In Situ Hybridization (FISH)cytogenetics showed (low grade B cell lymphoma) negative, and molecular MYD88 mutation negative. Peripheral blood flow cytometry showed the same cell population as bone marrow (Figure 3). Hairy cell leukemia was ruled out due to the lack of CD11c and negative BRAF mutation testing. Lymphoplasmacytic lymphoma** **was ruled out due to a negative MYD88 mutation. Hepatitis C antibody testing was negative, and no other cytopenias, except for anemia related to her warm autoimmune hemolytic anemia, were seen on work-up. Overall, the phenotypic and genetic testing, flow cytometry, and bone marrow results, as well as splenomegaly on imaging were suggestive of a diagnosis of splenic marginal zone lymphoma with Intergruppo Italiano Linfomi (IIL)** **prognostic index 2.

At that time, the decision was made to treat her warm autoimmune hemolytic anemia with prednisone. She remained steroid dependent for 7 months with decreases in the hemoglobin concentration when steroids were weaned. Due to persistent cytopenia in the setting of splenic marginal zone lymphoma, the decision was made to treat her with four cycles of rituximab. After treatment with rituximab, her hemoglobin normalized without signs of hemolysis, and spleen size decreased on CT imaging (13.4 cm in AP diameter) (Figure 1B), consistent with the treatment response. 

She remained symptom-free with no evidence of disease for 15 months status post rituximab treatment, before presenting again with airway edema requiring hospital admission. Shortly afterwards, she saw an allergist as an outpatient, and labs showed undetected complement component C1Q and low levels of C1 esterase inhibitor activity, C1 esterase inhibitor protein, and CH50 (Table 3). Low levels of these completed pathway enzymes are seen in acquired angioedema due to the consumption of these proteins and lead to an unregulated release of bradykinin and other vasoactive peptides. Based on these labs and her prior history she was diagnosed with AAE given her age at first presentation and the underlying SMZL. 

**Table 3:** Immunology Labs After Recurrent Angioedema

Lab	Reference value	Patient's level at first admission
Complement component C1Q	5.0-8.8 mg/dl	undetected
C1 esterase inhibitor level activity	>= 68%	28%
C1 esterase inhibitor protein	21-39 mg/dl	<3 mg/dl
CH50	31-60 U/ml	<10 U/ml

Approximately 3 months later, she presented with angioedema. CT imaging showed worsening splenomegaly (18 cm in AP diameter, enlarged from 13.4 cm on prior imaging (Figure 1B)). Peripheral blood flow cytometry showed abnormal lymphocytes (CD19+, CD20+, CD5-, CD10-), and elevated LD 654 U/L (reference range < = 250 U/L) consistent with relapsed SMZL. She was treated with rituximab while inpatient, with the resolution of her symptoms and splenomegaly. 

Unfortunately, on routine monitoring 7 months later, CT showed enlarging spleen size and new lymphadenopathy in the left inguinal and bilateral iliac region. She subsequently developed angioedema symptoms again and was again found to have a recurrence of her splenic marginal zone lymphoma. She was started on bendamustine and rituximab for six cycles with curative intent according to the National Comprehensive Cancer Network (NCCN) ​[9]. She has regular follow-ups with oncology, and allergy and immunology for ongoing surveillance and treatment. 

At the time of writing this manuscript, she is doing well and is currently in the middle of six cycles of bendamustine and rituximab combined therapy. She is taking tranexamic acid 1300 mg twice daily for angioedema prophylaxis and has not had a recurrence of angioedema symptoms. Overall, she is tolerating her treatments well.

## Discussion

Here, we present a case of a patient with acquired angioedema that was ultimately found to have underlying splenic marginal zone lymphoma. This case underscores the critical importance of differentiating between AAE and hereditary angioedema at initial presentation, as the management and prognosis differ. In patients presenting with angioedema, initial immunology workup should include C1-INH level, C1-INH function, C4, C1q, and Anti-C1 INH antibody levels to differentiate between hereditary and acquired forms of angioedema [7]. C1-INH is a protease inhibitor that inhibits complement proteins [2]. When C1-INH is suppressed or is low it leads to an increased release of bradykinin and vasoactive peptides causing symptoms of angioedema [2]. In both types of angioedema, C1-INH levels can be normal or low, C1-INH function will be low, and C4 levels will be low. In AAE, the C1q level is low but will be normal in hereditary angioedema [7]. Additionally, in AAE, the anti-C1-INH antibody may or may not be present [7]. However, in hereditary angioedema, the Anti-C1-INH antibody is never detected [7]. On this patient’s initial presentation with angioedema, she was tested for C1-INH level and function and C4, which were all low. Of note, the C1q level was not tested, and she was initially given a presumed diagnosis of hereditary angioedema, despite her age at presentation.

Acquired angioedema is often associated with B cell lymphoproliferative disorders, autoantibodies against C1-INH, or both [1,2]. One of the most common lymphoproliferative disorders associated with AAE is SMZL [6,7]. The median age of diagnosis of splenic marginal zone lymphoma is 65 years but can occur as early as the third decade of life [6]. There is a slight female predominance, and it often presents asymptomatically, making it a challenging diagnosis [10]. Up to 10-15% of cases of SMZL are associated with hemolytic anemia or other autoimmune diseases [6]. SMZL is also associated with hepatitis C, and all patients should be screened for this, as treatment of hepatitis C can often treat the underlying lymphoma [10]. Indications for treatment of SMZL include constitutional symptoms, cytopenias including autoimmune anemia, symptomatic splenomegaly, progressive nodal disease, or the presence of hepatitis C infection [10]. In patients positive for hepatitis C, treatment with antivirals will often treat their lymphoma. In patients without hepatitis C, mainstays of treatment for splenic marginal lymphoma include observation for indolent cases, rituximab with or without chemotherapy, or the immunomodulating drug lenalidomide, and splenectomy (which has largely fallen out of favor) [6,11]. 

Marginal zone lymphomas are a type of B cell-derived non-Hodgkin lymphomas that include extra-nodal marginal cell lymphoma, nodal marginal zone lymphoma, and splenic marginal zone lymphoma [3]. Marginal zone lymphomas all share common somatic genetic mutations, including trisomies of chromosomes 3 and 18, deletions at 6q23, and defects in the nuclear factor kappa B (NFkB) and KLF2 pathways [3,6]. The cells in SMZL are CD5/CD10-negative, which can make the diagnosis challenging, given the differential for CD5/CD10-negative lymphomas includes hairy cell leukemia, lymphoplasmacytic lymphoma (LPL), marginal zone lymphoma, and CD5-chronic lymphocytic leukemia [12]. SMZL can be differentiated from hairy cell leukemia, which is often strongly positive for CD11c, CD25, and CD103, while these markers are all negative in SMZL [12]. In addition, BRAF mutation is also positive in hairy cell leukemia and negative in SMZL. MYD88 mutation is a defining mutation in LPL and is often negative in SMZL [12]. Our patient had a negative MYD88 mutation on molecular cytogenetics, again leading to a final diagnosis of SMZL. Splenic rupture, as observed in other hematological malignancies such as chronic myeloid leukemia, can occur in conditions like SMZL, given shared features of splenomegaly and immune dysregulation [8]. Close monitoring and patient education about potential symptoms are crucial to ensure timely intervention and prevent life-threatening complications. 

After multiple episodes of angioedema due to underlying SMZL, work-up for warm autoimmune hemolytic anemia and splenomegaly lead to a diagnosis of SMZL. Early initiation of treatment for underlying lymphoproliferative disease could potentially help prevent recurrent attacks. At times, the diagnosis of AAE can be challenging, and clinicians need to have a high index of suspicion when patients present with angioedema later in life. Upon initial diagnosis of AAE, it is recommended that the following work-up is completed: complete blood count with differential (CBC w/diff), C4 level, C1-INH level and function, C1q level, quantitative immunoglobulins, serum and urine immunofixation, full-body CT, and bone marrow biopsy [13]. 

If AAE in this patient** **had been identified earlier and treated, it may have reduced some of her angioedema attacks, as multiple studies have shown reductions in angioedema following the appropriate treatment of an underlying lymphoma [5,14]. In addition, the literature suggests that if an initial underlying lymphoproliferative or autoimmune diagnosis is not found when a patient is diagnosed with acquired angioedema, routine yearly screening, including age-appropriate cancer screening, CBC w/diff, serum protein electrophoresis, chest X-ray, abdominal ultrasound, and complete physical exam, should be conducted as patients can develop a lymphoproliferative disease years after the initial diagnosis of angioedema [5,14,15,16] This case is further unique in that the recurrence of her SMZL was detected after recurrent symptoms of angioedema; it suggests that if a patient presents with recurrent angioedema, recurrence of underlying lymphoproliferative disorder should be considered. This case highlights the importance of a thorough work-up, often requiring the collaboration of an oncologist and allergist, in the care of a patient who presents with angioedema for the first time after the third decade of life.

## Conclusions

The exact mechanism of how AAE and lymphoproliferative disorders are linked is yet to be determined and is an active area of research. However, the association is strong enough that when a patient presents with angioedema for the first time, it is important to obtain proper labs to distinguish between hereditary and AAE. If it is determined that the patient has acquired angioedema, additional work-up looking for lymphoproliferative or autoimmune disorders should be done at initial diagnosis as well as yearly afterwards if an underlying disease is not initially noted due to the high association with lymphoproliferative disorders. A particularly high clinical suspicion should be had for the development of SMZL as this is the most common lymphoproliferative disorder associated with AAE, and the assistance of an oncologist is likely warranted given the complex diagnostic studies required to make a definitive diagnosis of SMZL. Early diagnosis, treatment, and regular surveillance of underlying lymphoproliferative disorders may prevent subsequent angioedema attacks. Accordingly, if a patient presents with recurrent angioedema, recurrence of underlying lymphoproliferative disorder should be considered. Further research is needed to better understand the mechanism of how acquired angioedema and lymphoproliferative disorders are interlinked, which could lead to additional testing and therapeutics to help treat and prevent angioedema attacks and treat underlying disorders. 
